# Pulmonary *Talaromyces marneffei* misdiagnosed as smear‐negative pulmonary tuberculosis

**DOI:** 10.1111/crj.13661

**Published:** 2023-07-06

**Authors:** Xiaojuan Chen, Jiwei Zhao, Lin Chen, Yanxia Chen, Jinlin Liu

**Affiliations:** ^1^ Department of Laboratory Medicine The Second Affiliated Hospital of Hainan Medical University Haikou China; ^2^ Department of Laboratory Medicine Nanjing Lishui District Hospital of Traditional Chinese Medicine Nanjing China; ^3^ Department of Rheumatology and Immunology, South China Hospital, Medical School Shenzhen University Shenzhen China; ^4^ Department of Clinical Laboratory, South China Hospital, Medical School Shenzhen University Shenzhen China

**Keywords:** *Talaromyces marneffei*, tuberculosis

Dear editor

The global burden of tuberculosis (TB) is escalating and has become a major health challenge. Acid‐fast bacilli smear‐negative TB patients are the major source of TB transmission to healthy individuals when left untreated.^1^ Herein, we present a case of pulmonary *Talaromyces marneffei* misdiagnosed as smear‐negative pulmonary tuberculosis and treated with antituberculosis drugs for 1 year, highlighting the importance of detailed morphological information on Gram staining and fungal culture of the sputum.

A 47‐year‐old Chinese male farmer presented to a respiratory outpatient clinic with a 1 year history of recurrent cough, expectoration, and hemoptysis for 1 day. He had been clinically diagnosed with pulmonary tuberculosis 1 year prior based on the clinical symptoms and typical lung CT image, but without *Mycobacterium tuberculosis* or other microorganisms isolated from the blood, sputum, and bronchoalveolar lavage fluid (BALF), and no tumor cells were found. His symptoms had not significantly improved despite antituberculosis drug and antibiotic treatment (isoniazid, rifapentine, ethambutol, and moxifloxacin) in the preceding year, and received bronchial artery embolization twice to treat hemoptysis at our hospital. Moreover, he had no fever, hepatosplenomegaly, or intestinal or skin symptoms during this year. Before the diagnosis of pulmonary tuberculosis, the patient was healthy and immunocompetent and had no chronic obstructive pulmonary disease, diabetes, immune deficiency, or travel history.

Upon admission, lung CT revealed a cavity (red arrow), an inflammatory granuloma‐like area (yellow arrow), exudation (blue arrow) (Figure 1A), and an enlarged mediastinal lymph node (red arrow) (Figure 1B), which was highly suggestive of pulmonary tuberculosis. Further laboratory examination revealed normal hematological and biochemical test results, negative interferon‐gamma release assays and tumor cell markers, and no acid‐fast bacilli were identified on the smear. In addition, bacterial cultures of sputum and blood, *Aspergillus galactomannan*, and HIV tests were negative.

**FIGURE 1:**
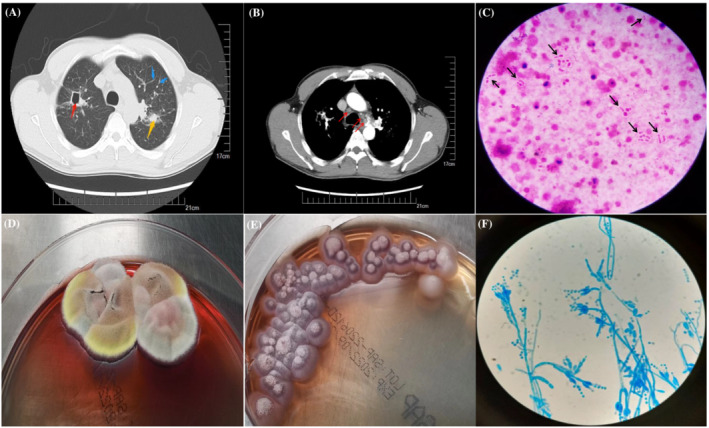
A case of pulmonary *Talaromyces marneffei* misdiagnosed as smear‐negative pulmonary tuberculosis. (A) The lung CT of this patient revealed a cavity (red arrow), inflammatory granuloma like area (yellow arrow), exudation (blue arrow), (B) and enlarged mediastinal lymph node (red arrow). (C) Gram staining of asputum smear revealed dachshund‐like yeast‐like cells with acentral septum (black arrow). The sputum culture at 25°C (D) or 35°C (E) after 5 days. (F) Lactophenol cotton blue staining of the sputum culture after 4 days.

Gram staining of the sputum smear showed numerous dachshund‐like, yeast‐like cells with a central septum (black arrow) (Figure 1C), raising the suspicion of fungal infection. Further fungal culture of the sputum showed the fungus growing in a mold form arranged in a mulberry‐like pattern at 25°C (Figure 1D) or 35°C (Figure 1E) after 5 days of culture. Moreover, lactophenol cotton blue staining confirmed the presence of numerous mold‐like organisms in the sputum culture after 4 days (Figure 1F). Macrogenome sequencing of the BALF confirmed *Talaromyces marneffei* infection. The patient was then administered itraconazole, and the respiratory symptoms resolved rapidly with no recurrence after 4 months of follow‐up.

Talaromycosis is an invasive mycosis endemic to tropical and subtropical Asia, caused by the pathogenic fungus *Talaromyces marneffei*. Approximately 17 300 cases are diagnosed annually, and despite one third of the diagnosed cases resulting in death, it has received little attention worldwide.^2^ It primarily affects HIV and other immunocompromised patients and disproportionally affects agricultural workers in rural areas. It is also strongly associated with the tropical monsoon season, where flooding and cyclones can exacerbate this disease.^3^ *Talaromyces marneffei*, a dimorphic fungus that appears as a mold at 25°C and as a yeast at 37°C, is an important opportunistic pathogen that can cause life‐threatening systemic infections. Herein, we have reported on a *Talaromyces marneffei* infection case that presented with recurrent cough and expectoration. Lung CT strongly suggested pulmonary tuberculosis and was misdiagnosed as pulmonary tuberculosis in clinics, even after 1 year of antituberculosis drugs in this patient. Thus, this case illustrates the importance of obtaining detailed morphological information from Gram staining under light microscopy and further fungal cultures of sputum or BALF. Pulmonologists should also consider *Talaromyces marneffei* infection as a possible cause of recurrent cough and expectoration in clinics, especially in endemic areas, or in non‐endemic areas when dealing with travel‐related cases, thus avoiding misdiagnosis and inappropriate treatment.

## AUTHOR CONTRIBUTIONS

Xiaojuan Chen and Lin Chen provided the picture and guided the clinical diagnosis. Yanxia Chen and Jiwei Zhao participated in the clinical diagnosis and analyzed the data. Jinlin Liu designed the study, analyzed the data and wrote the manuscript.

## CONFLICT OF INTEREST STATEMENT

We declare no competing interests.

## ETHICS STATEMENT

This study has been approved by the ethical committee of the Second Affiliated Hospital of Hainan Medical University. The patient provided written consent for the publication of this Clinical Picture.

## CONSENT FROM ALL AUTHORS

All authors reviewed this manuscript and agreed to submit this manuscript.

## Data Availability

Data sharing is not applicable to this article as no new data were created or analyzed in this study.

## References

[1] Mustafa H , Shah NN , Shahnawaz M , Yousuf M . Role of Gene Xpert in smear negative pulmonary tuberculosis. Indian J Tuberc. 2022;69(4):552‐557. doi:10.1016/j.ijtb.2021.08.035 36460388

[2] Wang F , Han R , Chen S . An overlooked and underrated endemic mycosis‐talaromycosis and the pathogenic fungus *Talaromyces marneffei* . Clin Microbiol Rev. 2023;36(1):e0005122. doi:10.1128/cmr.00051-22 36648228 PMC10035316

[3] Narayanasamy S , Dat VQ , Thanh NT , et al. A global call for talaromycosis to be recognised as a neglected tropical disease. Lancet Glob Health. 2021;9(11):e1618‐e1622. doi:10.1016/S2214-109X(21)00350-8 34678201 PMC10014038

